# Point-of-Care Sonographic Findings in Acute Upper Airway Edema

**DOI:** 10.5811/westjem.2016.9.31528

**Published:** 2016-10-04

**Authors:** Michael Schick, Kendra Grether-Jones

**Affiliations:** University of California, Davis, School of Medicine, UC Davis Medical Center, Department of Emergency Medicine, Sacramento, California

## Abstract

We describe a case where a patient presented with acute angiotensin-converting enzyme inhibitor (ACE-I) induced angioedema without signs or symptoms of upper airway edema beyond lip swelling. Point-of-care ultrasound (POCUS) was used as an initial diagnostic test and identified left-sided subglottic upper airway edema that was immediately confirmed with indirect fiberoptic laryngoscopy. ACE-I induced angioedema and the historical use of ultrasound in evaluation of the upper airway is briefly discussed. To our knowledge, POCUS has not been used to identify acute upper airway edema in the emergency setting. Further investigation is needed to determine if POCUS is a sensitive and specific-enough tool for the identification and evaluation of acute upper airway edema.

## INTRODUCTION

Angiotensin-converting enzyme inhibitor (ACE-I) induced angioedema is a known side effect of ACE inhibitors. This class of medication causes nonimmunoglobulin E mediated angioedema that is precipitated by bradykinin release. This type of angioedema is usually gradual in onset without rash and can involve various parts of the body. Its incidence is estimated to be 0.3–0.68%. Although the numbers appear small, due to the large number of patients taking these medications it is a common cause of angioedema. Symptoms may develop within hours of starting the medication or may take years to develop and typically involves the face, lips, tongue, and may involve the gastrointestinal mucosa.^1^

Patient presentations can be dramatic with significant facial swelling, voice changes, and critical airway compromise that requires immediate airway intervention. However, presentations can also be mild with subtle findings that do not clearly indicate the need for airway intervention. During these presentations it may not be readily apparent which patients require immediate intervention for rapid progression of airway edema, which can be monitored for airway compromise, and which can be safely discharged home. The current tools to evaluate the upper airway beyond physical exam include direct and indirect laryngoscopy, which involves equipment that may not be readily available in all settings, time for sedation and anesthesia, and patient discomfort.

## CASE REPORT

A 70-year-old female presented to the emergency department (ED) with shortness of breath and left upper and lower lip swelling. The night prior to presentation, the patient felt well without any complaints or issues. She woke up with a feeling of fullness in her lip, but denied visible swelling. Over the next two hours she had onset of left-sided lip swelling. The patient denied other facial swelling or feeling of difficulty swallowing. She also denied chest pain, abdominal pain, nausea, vomiting, fevers, and chills. She had never had similar symptoms previously.

Her past medical history included hypertension and one of her home medications was Lisinopril. Her uvula was resected many years ago due to sleep apnea, but she had no other relevant surgical, social or family history. Vital signs at presentation were temperature 36.7° C, blood pressure 128/80, heart rate 66, respiratory rate 18, and pulse oximetry was 100% on room air. On exam, left-sided edema and fullness to the upper and lower lip was present without tongue swelling. Her uvula was resected, but there was no posterior oropharyngeal swelling, stridor or muffled voice. The remainder of the exam was unremarkable, including no tachycardia, adventitious breath sounds, abdominal tenderness, rash or lower extremity edema. Further testing included labs and a chest radiograph, which were unremarkable.

**Figure 1:**
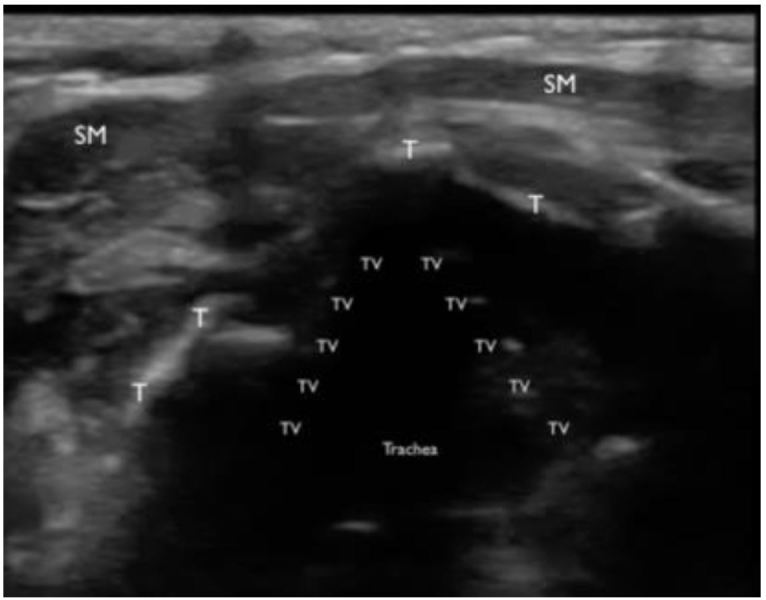
A transverse still image of the upper airway using the high frequency, linear transducer at the level of the true vocal cords (TV). Sternocleidomastoid muscles (SM) are seen anterior in the image. The true vocal cords appear as a linear structure that moves with phonations and is generally hypoechoic compared to the false vocal cords. The thyroid cartilage (T) appears hyperechoic in the image and provides a good acoustic window to visualize the vocal cords.

**Figure 2:**
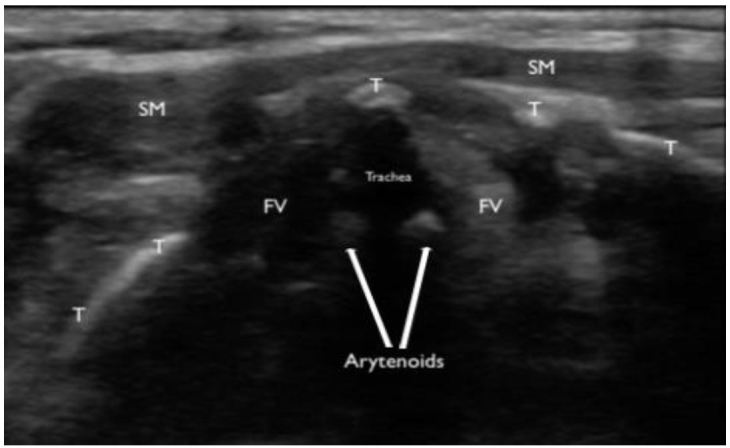
A transverse still image of the upper airway taken with a high frequency, linear transducer at the level of the arytenoid cartilage. Sternocleidomastoid muscle (SM) and thyroid cartilage (T) are re-demonstrated. The false vocal cords (FV) are visualized with noted asymmetry, edema on the patient’s left side (right side of the image).

**Figure 3:**
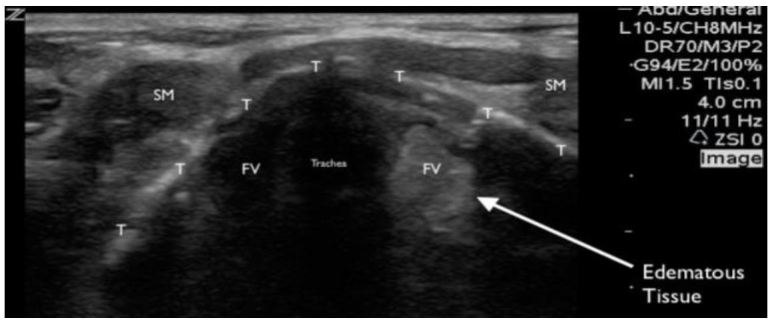
A transverse still image of the upper airway using the high frequency, linear transducer at the level just cephalad of the vocal cords and arytenoids. Asymmetry is identified with a hyperechoic fullness of the left side of the airway (right side of the image). This was immediately confirmed as subglottic edema at the level just cephalad of the vocal cords by indirect fiberoptic laryngoscopy.

**Figure 4:**
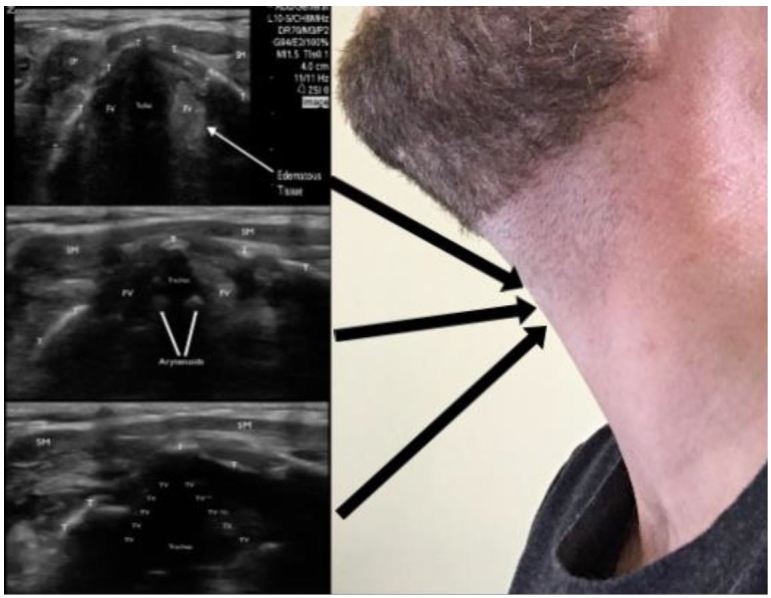
The previous three figures shown at their relative anatomic location. The transducer is held in the transverse plane with the indicator to the patient’s right side.

A bedside, point-of-care ultrasound (POCUS) was performed with the intention to evaluate the subglottic regions near the vocal cords for signs of airway edema. Images were acquired while using a Zonare ultrasound machine and a high frequency, linear transducer (10–15 MHz) in the soft-tissue exam setting. The patient was seated and was instructed to place herself in a sniffing position. Starting in the submandibular region and ending at the base of the anterior neck, serial transverse videos clips were obtained. Specific focus centered on the region above and below the thyroid cartilage. The vocal cords were identified and confirmed by patient phonation (Figure 1). Mild vocal cord asymmetry was noted with fullness on the left side as the transducer was slid cephalad to the level of the arytenoids (Figure 2). Significant asymmetry was noted at the region just cephalad to the vocal cords on the left side at the level of the false vocal cords. The subglottic region on the left appeared larger, more echogenic, with a distinct centrally demarcated mass that was not present on the patient’s right side (Figure 3). This was suspected to represent subglottic edema on the left side at the level of the false vocal cords. This was immediately confirmed by indirect fiberoptic laryngoscopy. Figure 4 demonstrates the relative anatomical location of each captured image at the level of the vocal cords, arytenoids, and false vocal cords. Video 1 is a narrated video demonstrating relevant anatomy, appearance on ultrasound, and identification of pathology.

Nasopharyngeal laryngoscopy (NPL) was performed approximately one hour after arrival to assess for vocal cord involvement. The NPL scope showed left-sided false vocal cord, true vocal cord and epiglottic swelling as well as edema of surrounding tissues. Her vocal cords were fully mobile without right-sided swelling. She was treated with diphenhydramine, famotidine and dexamethasone in the ED. For airway monitoring, the patient was admitted to the intensive care unit (ICU) for observation without immediate intubation.

## DISCUSSION

Ultrasound has been used for over two decades in the evaluation of the vocal cords and the upper airway.^2^,^3^ As a comfortable, non-invasive test ultrasound has been used to evaluate for vocal cord paralysis and lesions in both pediatric and adult patients.^4^–^6^ Ultrasound has also been used to evaluate for postextubation laryngeal edema. Measuring the air column width (ACW) during endotracheal tube balloon deflation has been found to be effective in ruling out mild to moderate laryngeal edema and similar to the cuff leak test.^7^–^9^ Vocal cord examinations use high-frequency transducers (8–15MHz) and typically have patients in a supine position with the neck slightly extended. Extension of the neck may increase the spaces between tracheal cartilage and broaden the acoustic window. Alternatively, patients may sit upright and be in the “sniffing position” with the head extended and neck slightly flexed. The patient should breathe spontaneously. External identification of the thyroid cartilage by physical exam should guide placement of the ultrasound transducer in the transverse plane at the level of the thyroid cartilage. The transducer is then slid superiorly and inferiorly in the transverse plane until the vocal cords are clearly identified. Evaluation of the vocal cords should occur with phonation by the patient using the “long E” for best visualization.^10^ This region, along with the regions above and below the vocal cords from the submandibular region to the base of the neck, should be interrogated for asymmetry.

In this case, the patient was observed for 24 hours in the ICU. Approximately 12 hours after initial presentation her swelling was mildly improved. She was treated supportively with scheduled diphenhydramine, solumedrol, and famotidine. Repeated ultrasound or fiberoptic laryngoscopy was not performed. The next morning the patient’s swelling had significantly improved and she was diagnosed with angioedema secondary to ACE inhibitors. She was discharged with instructions to stop Lisinopril as well as to complete a four-day course of prednisone and famotidine. After follow up with her primary care physician, no adverse events were reported. During hospitalization she noted that for approximately one week, she had been taking a different color Lisinopril than she was previously taking.

To our knowledge, POCUS has not been used to identify acute upper airway edema in the emergency setting. POCUS is fast, non-invasive and readily available in most EDs in the United States. POCUS accurately identified airway edema in this patient that did not have obvious symptoms or signs on history or physical exam. Color flow was not used in this case, but might be an adjunct in the evaluation of edematous tissue. Further investigation is needed to determine if POCUS is a sufficiently sensitive and specific tool for the identification and evaluation of acute upper airway edema.

## Figures and Tables

**Video:** A narrated video explaining the relevant anatomy, appearance on ultrasound, and identification of pathology.
